# Parent-Offspring Conflict and the Persistence of Pregnancy-Induced Hypertension in Modern Humans

**DOI:** 10.1371/journal.pone.0056821

**Published:** 2013-02-25

**Authors:** Birgitte Hollegaard, Sean G. Byars, Jacob Lykke, Jacobus J. Boomsma

**Affiliations:** 1 Centre for Social Evolution, Department of Biology, University of Copenhagen, Copenhagen, Denmark; 2 Department of Obstetrics and Gynaecology, Roskilde Hospital, Roskilde, Denmark; Indiana University, United States of America

## Abstract

Preeclampsia is a major cause of perinatal mortality and disease affecting 5–10% of all pregnancies worldwide, but its etiology remains poorly understood despite considerable research effort. Parent-offspring conflict theory suggests that such hypertensive disorders of pregnancy may have evolved through the ability of fetal genes to increase maternal blood pressure as this enhances general nutrient supply. However, such mechanisms for inducing hypertension in pregnancy would need to incur sufficient offspring health benefits to compensate for the obvious risks for maternal and fetal health towards the end of pregnancy in order to explain why these disorders have not been removed by natural selection in our hunter-gatherer ancestors. We analyzed >750,000 live births in the Danish National Patient Registry and all registered medical diagnoses for up to 30 years after birth. We show that offspring exposed to pregnancy-induced hypertension (PIH) in trimester 1 had significantly reduced overall later-life disease risks, but increased risks when PIH exposure started or developed as preeclampsia in later trimesters. Similar patterns were found for first-year mortality. These results suggest that early PIH leading to improved postpartum survival and health represents a balanced compromise between the reproductive interests of parents and offspring, whereas later onset of PIH may reflect an unbalanced parent-offspring conflict at the detriment of maternal and offspring health.

## Introduction

Relative to the great apes, humans have long pregnancies and relatively high perinatal risks for mother and offspring. This condition is generally perceived as being due to selection for large fetal brains and constraints on evolutionary responses in the pelvic bones of adult females because of trade-offs with efficient bipedal locomotion [1]. Caesarian section and hygienic obstetric practices have now greatly reduced the risks of giving birth in affluent societies, and considerable progress has been made in developing countries. However, advances in understanding pregnancy complications related to elevated maternal blood pressure, such as preeclampsia and eclampsia, have been less impressive. These conditions remain a major cause of maternal and infant morbidity and mortality, especially in developing countries [2]. Preeclampsia is a multi-organ disease affecting liver, kidneys and the central nervous system and can in severe cases progress to organ failure (eclampsia). At present, there is no cure except for inducing birth, often prematurely [3].

While it seems relatively straightforward to explain the narrow human birth canal as an unavoidable evolutionary constraint, this kind of interpretation seems invalid for hypertension-induced problems during pregnancy. It would seem incomprehensible that the fetus harms its pregnant mother while being so intimately and obligatorily dependent on maternal health, both before and after birth. And even if any genetic tendencies for fetuses to increase maternal blood pressure would have arisen during our evolutionary past, it seems puzzling why natural selection has not removed such fitness-reducing traits in our hunter-gatherer ancestors. The alternative explanation that preeclampsia and other hypertension-related pregnancy complications are mostly a modern affluent society problem does not hold either, because preeclampsia is also prevalent in developing countries where there is little access to modern health care [2], [4]. Despite many studies that have identified proximate mechanisms in the etiology of preeclampsia [2], [5]–[7], much of the causal variation for hypertensive disorders of pregnancy remains unknown, suggesting that this health problem also needs a broader explanation. Such explanations can take two non-mutually exclusive forms in that they can focus either on the proximate physiological mechanisms, or on the evolutionary forces of natural selection that have shaped human pregnancies. In this paper we use the latter type of approach.

Parent-offspring conflict (POC) theory [8] hypothesizes that it is in the genetic interests of a focal offspring to try – within limits – to extract more resources than the mother is selected to provide, and in her interest to provision her resources more equally between current and future offspring. Many autosomal genes have been implicated as being involved in POC interactions [9]–[11], but also imprinted genes may be involved in the pathogenesis of hypertensive disorders of pregnancy [12]–[14]. The theory maintains that there is an interval of fetal-provisioning where the maternal and paternal interests are not aligned because the father of the focal fetus has a probability of less than 100% of also fathering the focal mother’s next child, owing to serial monogamy or varying degrees of promiscuity [12]. This implies that paternal genes expressed in the placenta have been under consistent selection to express phenotypes that somehow increase maternal blood pressure as this would generally enhance fetal resource provisioning via the mother-infant circulation interface [15], [16]. Such genes would be particularly likely to succeed if they would be able to silence the placental expression of maternal alleles, but that inevitably induces selection for opposite maternal imprints at complementary loci because a mother is symmetrically related to all her children. In normal pregnancies these maternal/paternal-specific imprinting effects would be expected to reach a balance that produces a healthy offspring while preserving maternal health. A substantial number of imprinted genes with opposite parent-of-origin effects on embryo provisioning are now known, both in humans and in mice [14], [17], [18] and other studies show that disruptions to the balance of these genomic imprints can have severe health consequences [19]–[22].

**Figure 1:**
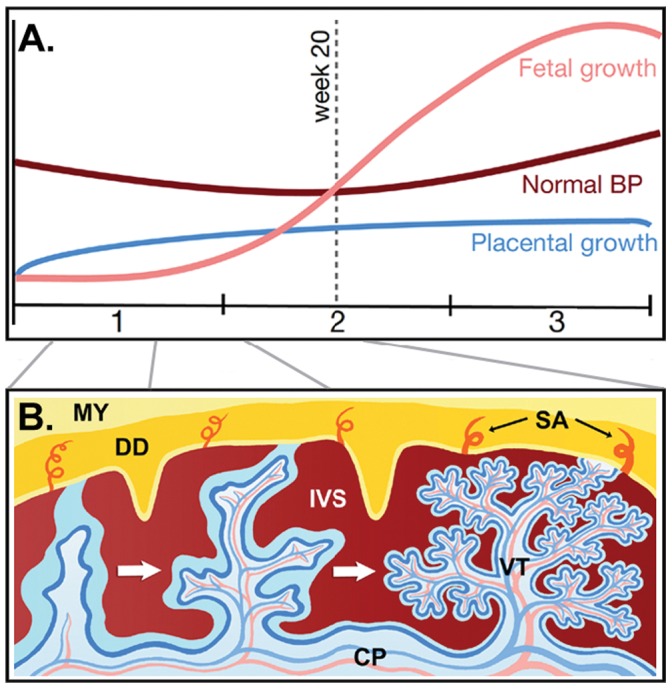
Typical patterns of placental-fetal growth, maternal blood pressure and tissue interactions across the ∼40 weeks (i.e. trimesters 1,2 and 3) of human pregnancy. (**A**) Typical curves of placental growth (blue), fetal growth (pink) and normal maternal blood pressure (brown), approximated based on various sources [43]–[46]. (**B**) Schematic diagram of trophoblast invasion (up to week 20–22) that upscale fetal provisioning via the placental blood vessels and tissues that are either 100% maternal (yellow/orange shades) or 50–50 maternal and paternal and thus genetically identical to the offspring (blue shades) and where paternally imprinted genes can be expressed. Maternal structures: MY: myometrium, DD: decidua or uterine lining during pregnancy, SA: spiral arteries, IVS: intervillous space (contains pool of maternal blood; red coloured). Fetal structures: CP: chorionic plate, VT: villous tree (growth of VT’s (from left to right) is completed by week 20–22). The genomic imprinting hypothesis for PIH assumes that paternally imprinted genes expressed in the blue tissues can induce enhanced maternal blood pressure via physiological and morphological adjustments [15], unless maternally expressed genes in the yellow/orange tissues induce compensating phenotypic effects to match this fetal demand for increased resource provisioning [47].

General explanations for hypertensive disorders of pregnancy associated with POC would be most compelling if it could be shown that milder forms such as pregnancy-induced hypertension (PIH) are beneficial for offspring health, as that would imply that the overall benefits of hypertension during pregnancy may balance or even exceed the negative costs of the more severe forms such as preeclampsia, rather than preeclampsia imposing a population-level genetic load. Several studies have demonstrated negative effects of hypertensive disorders on developing fetuses and newborns [23]–[27] and on subsequent offspring health [28], [29] in spite of increases in birth weight [16], but outcomes are not always pathological. Although not the focus of earlier studies, Symonds [30] found lower average perinatal mortality of babies born to mothers with PIH, and others reported improved health in offspring exposed to any kind of hypertensive disorder of pregnancy (i.e. after pooling PIH, preeclampsia, eclampsia), particularly in small for gestational age neonates [31], [32]. However, no previous studies have focused on PIH specifically while separating it from the various milder and more severe forms of hypertensive pregnancy disorders in order to investigate perinatal mortality and the long-term impact on offspring health. Moreover, PIH throughout pregnancy may have different effects depending on when it occurs, due to changing fetal demands and natural background variation in the typical maternal blood pressure [16] (Fig. 1). The capacity of maternal blood supply (i.e. veins and arteries that connect fetal-maternal tissues, Fig. 1) is largely determined early in pregnancy (first 20–22 weeks) when placental invasion of the endometrium and modification of the spiral arteries takes place [12]. This suggests that the effects of PIH on maternal-fetal interactions and ultimately offspring health may vary depending on its occurrence and timing throughout pregnancy, yet no study has investigated this before. Here, we examine these relationships by utilizing the national health registries in Denmark, which provided us with data on >1.8 million births from 1977–2007 and diseases diagnosed within >5 million people from 1977–2009.

## Results

**Figure 2:**
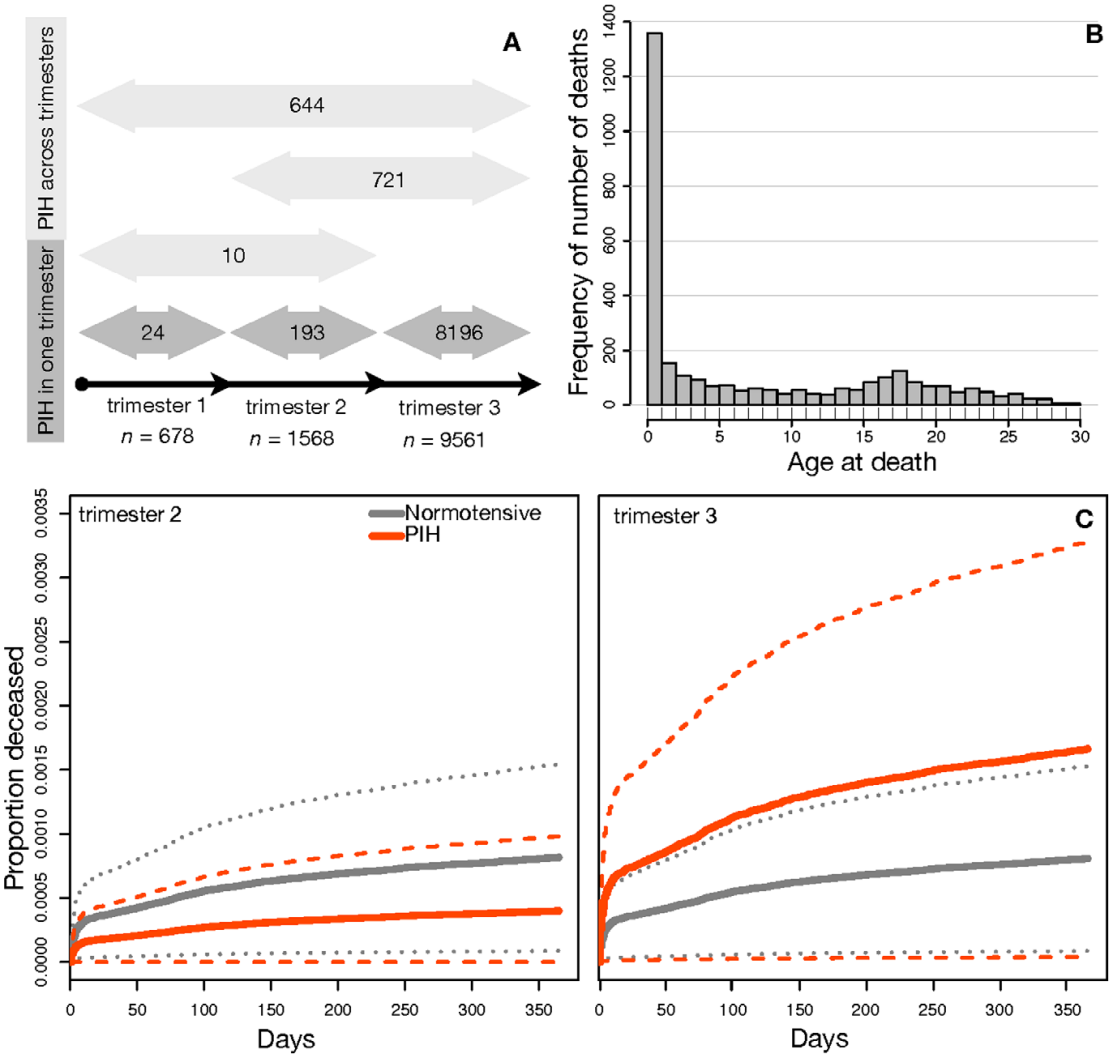
Classification of the distribution of women diagnosed with pregnancy-induced hypertension (PIH) included in our study **(A).** PIH either only occurred across one trimester (1, 2 or 3; dark grey arrows) or across multiple trimesters (1–2, 2–3, 1–3; light grey arrows). Numbers within arrows are sample sizes. Elevated blood pressure is registered as a specific diagnose code (Table S2) in the Danish National Patient Registry. As women’s diagnoses were registered in our database with multiple in and out dates, this classification was the most accurate possible. (**B**) Distribution of age at death across the first 30 years of life for Danish offspring born between 1977 and 2007. Deaths in total were 3,118, of which 34.51% are accounted for in the first year after birth, and 22.5% in the following 2–5 years of life. Mortality in the first year of life was mainly due to perinatal complications (21.2%) and congenital malformations (38.5%). (**C**) Kaplan-Meier survival plots of offspring mortality in the first year of life. Red lines represent cumulative mortality of offspring born to mothers who experienced pregnancy-induced hypertension (PIH) in trimester 2 (left panel) or 3 (right panel), relative to those with normotensive pregnancies (grey lines; identical in both plots). Lines represent averages (solid) ±95% confidence intervals (dashed) in trimester 2 (*P = *0.17) and in trimester 3 (*P*<0.001). For offspring born from pregnancies with PIH, the difference in risk of cumulative mortality between trimester 2 and 3 is significantly different (*P = *0.0028). Survival curves for trimester 1 could not be calculated as no offspring died who were born to mothers with PIH in trimester 1 (*n* = 678). Data cover the period of registration between 1979–2009.

Using Cox regression that adjusted for potentially confounding effects, we estimated the risk of infant mortality and diseases after birth, depending on the occurrence of maternal PIH by trimester (Fig. 2A) or preeclampsia. In the PIH analysis, three dummy variables coded for the continuous and intermittent presence or absence of elevated blood pressure in trimesters 1, 2 or 3. For example, the ‘PIH in trimester 1′ dummy variable (total PIH diagnoses = 678, Fig. 2A) included PIH diagnoses that started (light grey bars, *n* = 10 and *n* = 644) or only occurred (dark grey bar, *n* = 24) in trimester 1, whereas the ‘PIH in trimester 2′ dummy variable (total PIH diagnoses = 1568) included PIH diagnoses that started (light grey bar, *n* = 721), only occurred (dark grey bar, *n* = 193) or continued (light grey bars, *n* = 10 and *n* = 644) into trimester 2. This “overlapping” design allowed risk ratio comparisons between trimesters, while also adjusting for the effects of PIH that continued across multiple trimesters. This would not have been possible if we had only included the specific diagnoses for each of the trimesters (dark grey bars, Fig. 2A). Moreover, a trimester-specific analysis would have decreased sample sizes to the point of producing unreliable Cox analysis estimates (see Table S3), particularly for trimester 1. In the preeclampsia analysis, only one binary dummy variable was needed to code for its presence as this diagnosis predominantly occurred in trimester 3.

We found significant differences in infant mortality (Fig. 2B) depending on the trimester in which PIH occurred, relative to normotensive pregnancies. If PIH occurred in trimester 3, risk of infant mortality was doubled (hazard or risk ratio (RR) = 2.06, 95% CI = 1.37–3.09, *P*<0.001, Fig. 2C (right panel)), similar to the increased mortality risk experienced when more severe forms occur (i.e. preeclampsia, RR = 2.37, 95% CI = 1.27–4.44, *P* = 0.006). For PIH in trimester 2, the risk ratio was <1 indicating a lower risk of infant mortality. This difference was not significant when compared to normotensive pregnancies in the same trimester (RR = 0.44, 95% CI = 0.14–1.41, *P* = 0.17, Fig. 2C (left panel)), but was significant when compared to the survival curve for children born to mothers suffering from PIH in trimester 3 (*P* = 0.0028). All infants of the 678 mothers with PIH in trimester 1 survived the first year of life. This zero value for the risk of infant mortality precluded that formal Cox regression coefficients (risk ratio, *P*-value) could be obtained in comparison with normotensive pregnancies, but suggests that PIH-induced mortality is extremely low when it arises early in pregnancy. Mortality beyond the first year of life was not included as most infant mortality was observed during the perinatal period (Fig. 2B).

**Figure 3:**
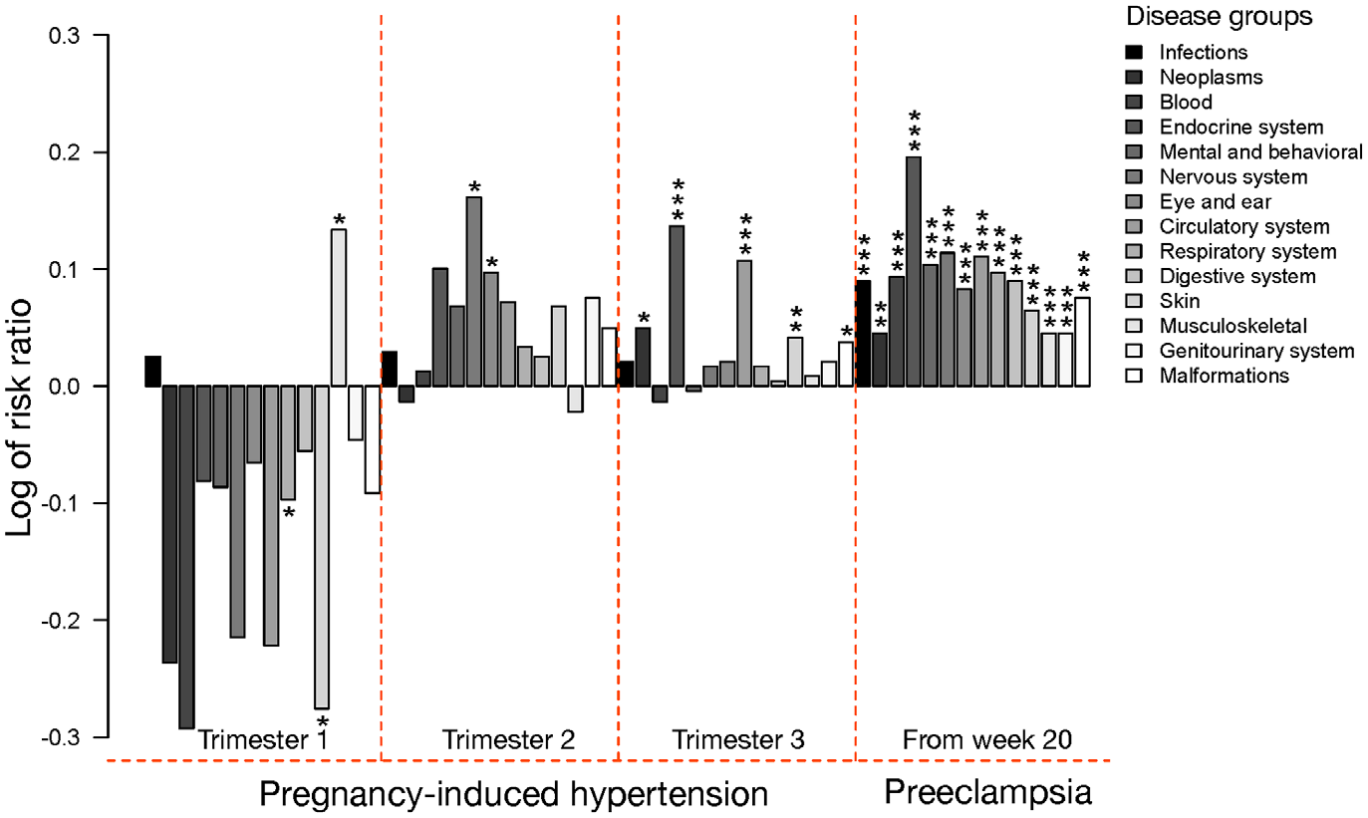
Risk of being diagnosed with a disease belonging to one of the 14 disease groups for offspring born to mothers with pregnancy-induced hypertension (PIH) in trimester 1, 2 or 3, or preeclampsia from week 20 onwards. Risk ratios and *P* values (*P<0.05, **P<0.01, ***P<0.001) come from Cox regressions and are relative to women with normotensive pregnancies (controls). Logged risk ratios represent increased (above 0) or decreased (below 0) risk of being diagnosed with disease across the ages of 1–27 years. The fourteen disease groups are listed towards the upper right with the same shading as in the bar plots. The plot is based on numbers provided in Table S1.

Our next objective was to estimate the risk of infant disease after birth with up to 27 years of follow up, depending on the occurrence of the same set of pregnancy-related complications as above. We found largely consistent changes in risk ratio direction for the 14 disease groups considered (Fig. 3, Table S1), depending on when or what type of hypertension occurred throughout the pregnancy. The highest risks for disease were observed when mothers had preeclampsia, with risk of being diagnosed significantly enhanced across all 14 disease groups in congruence with results from Wu *et al.* [28]. If PIH occurred in trimester 3 (but without advancing to preeclampsia), the direction of disease risk was also consistently positive (12 out of 14) suggesting a moderately increased overall risk for disease, with risks in five of the groups being significantly enhanced. This trend continued for PIH having been diagnosed in trimester 2, but with only two of the disease groups having significantly enhanced risk ratios compared to normotensive pregnancies.

**Figure 4:**
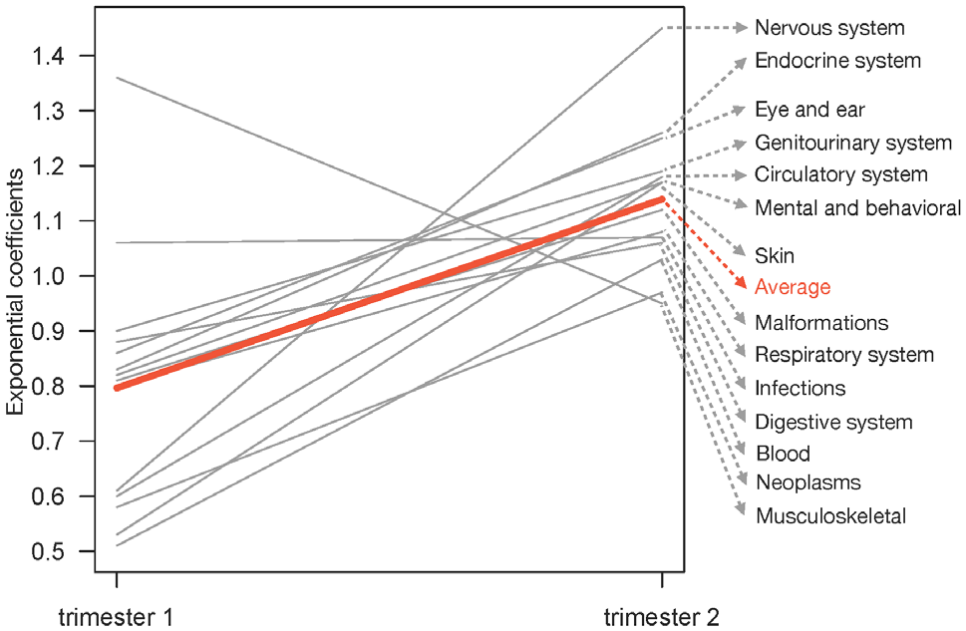
Interaction plot of the risk (exponentiated risk coefficients) of being diagnosed within the first 27 years of life for offspring born to mothers with pregnancy-induced hypertension (PIH) in either trimester 1 (left) or trimester 2 (right). Coefficients <1 indicate decreased risk and >1 increased risk of being diagnosed with disease. Grey lines represent the 14 main disease groups and the red line is the overall average, which was significantly higher (*P*<0.001) for offspring born to mothers with PIH in trimester 2 compared to trimester 1. The plot is based on numbers from Table S1.

In sharp contrast with the increased risk of most diseases due to hypertension in trimester 2 and 3, PIH occurring in trimester 1 appears to have the opposite effect on offspring health, with risk of disease being reduced in 12 of the 14 main disease groups, relative to normotensive pregnancies (Fig. 3). In spite of the rather modest number of PIH diagnoses that occurred in trimester 1 (*n* = 678; Fig. 2A), two of these were significant, including a decreased risk for skin (RR = 0.53, *P*<0.05) and respiratory (RR = 0.80, *P*<0.05) diseases (cf Table S1). This suggests that developing fetuses will only gain benefits of maternal hypertension if it starts early during pregnancy when the fetal-maternal connections in the placenta are being formed. We further used a meta-analysis approach where coefficients and standard errors from the 14 main Cox regressions were used in a Student t-test (for dependent samples) to compare whether PIH-in-trimester-2 risk ratios were significantly larger than PIH-in-trimester-1 risk ratios. This resulted in 14 *P* values, which were then combined into an overall *P* value using weighted-Z and Fisher’s approach in the survcomp package in R. This showed that the average disease risk difference between trimester 1 and 2 was highly significant (*P*<0.001 in all methods (Fig. 4)) confirming that PIH has vastly different effects on developing fetuses and the subsequent general health of children depending on timing of exposure.

## Discussion

Our results indicate significantly reduced first year mortality and later-life disease risks for offspring born to mothers with PIH early in pregnancy, whereas PIH in later trimesters increases these risks, so they come to approach those connected with preeclampsia when PIH occurs in trimester 3. A proximate explanation for this shift may be that fetuses that became endowed with slightly insufficient placentas have ways to counter the threat of remaining undernourished by increasing maternal blood pressure to drive more nutrients across the fetal-placental barrier [12]. This would be an expression of parent-offspring conflict (which also may apply to non-imprinted autosomal genes subject to POC), but one conditional on a suboptimal start in fetal life that would - from a paternal gene perspective - justify a shift in the provisioning balance between mother and child in spite of enhanced risks for both maternal and offspring health. As most fetal growth occurs in the last half of trimester 2 and in trimester 3 (and most placental growth in trimester 1, Fig. 1), such enforced catch-up growth would fit our observation that pregnancies with PIH result in somewhat (2.1%) lower average birth weight in comparison with normotensive pregnancies (3,469 versus 3,544 g, Student’s t-test *P*<0.001, Table 1). This average remains firmly within the range that is considered appropriate for gestational age, similar to the even lower average birth weight (3,361 g) of babies born to preeclamptic mothers (Table 1, Student’s t-test *P*<0.001 in comparison with the PIH cohort). While these birth weight differences are of some interest in their own right [16], they were included as covariates in our analyses and thus accounted for in the results presented in Figs. 2, 3, 4 and Table S1. The fetal under-nutrition hypothesis does therefore not explain why PIH in trimester 1 brings survival and later health benefits to children, relative to normotensive pregnancies.

**Table 1:** Baseline statistics of the parents and their offspring included in our study.

	normotensive	PIH	preeclampsia	all
number of births	704013	9788	44531	758524
**maternal**, *n*	367709	8607	38499	413594
age at delivery (years)	28.61 (4.63)	29.21 (5.11)	27.91 (5.11)	28.57 (4.67)
parity (sibling number)	1.68 (0.90)	1.67 (0.90)	1.53 (0.82)	1.68 (0.90)
BMI (2003 onwards)	23.79 (4.54)	26.61 (5.98)	26.15 (5.87)	23.92 (4.65)
smoking (1993 onwards), *n* (%)	29774 (4.23)	315 (3.22)	1789 (4.02)	31878 (4.20)
previous induced abortions	0.14 (0.44)	0.18 (0.49)	0.21 (0.54)	0.15 (0.45)
previous spontaneous abortions	0.13 (0.42)	0.18 (0.51)	0.17 (.0.49)	0.13 (0.42)
education, *n* (%)				
0–12 years	31246 (4.44)	547 (5.59)	2586 (5.81)	34379 (4.53)
12–15 years	114992 (16.33)	1811 (18.50)	9696 (21.77)	126499 (16.68)
15–17 years	283038 (40.20)	4089 (41.78)	18590 (41.75)	305717 (40.30)
17–20 years	215401 (30.59)	2740 (27.99)	11329 (25.44)	229470 (30.25)
20–22 years	56003 (7.95)	572 (5.84)	2210 (4.96)	58785 (7.75)
22+ years	3333 (0.47)	29 (0.30)	120 (0.27)	3482 (0.46)
**paternal**				
age at delivery	31.32 (5.56)	31.68 (5.81)	30.64 (5.92)	31.28 (5.59)
**maternal/paternal**				
combined annual income	201400	360000	334000	211500
**births**				
male, *n* (%)	360591 (51.22)	5026 (51.35)	23285 (52.29)	388902 (51.27)
female, *n* (%)	343422 (48.78)	4762 (48.65)	21246 (47.71)	369430 (48.70)
birth weight (grams)	3544 (508.62)	3469 (573.22)	3411 (685.73)	3535 (522.53)
gestational length (days)	279.3 (10.84)	277.8 (11.48)	275.0 (14.77)	279.1 (11.17)
BMI at birth	14.40 (2.01)	14.25 (1.98)	14.34 (2.04)	14.40 (2.01)
Apgar5 score	9.89 (0.61)	9.82 (0.77)	9.78 (0.78)	9.88 (0.62)

Maternal variables are divided into mothers with normotensive (control) pregnancies relative to those who experienced pregnancy-induced hypertension (PIH) or preeclampsia. Values are means (± SD) except where indicated otherwise. *P*-values from t-tests between normotensive and PIH/preeclampsia cohorts were all between *P* = 4.867e−14 and *P*<2.2e−16, except for parity differences between normotensive and PIH mothers, which were not statistically different (*P* = 0.135). All data are based on records of the Danish National Patient Registry, spanning the years 1979–2007. Apgar5 score is an evaluation of the neonate’s health five minutes after birth on a scale from 0 to 10, where 10 indicates best possible health. Woman with PIH were on average slightly older, had more previous spontaneous and induced abortions, higher family income, and higher BMI compared to the rest of the sample. These variables were all included in the Cox regressions so their effects were partialled out. Because BMI was only recorded from 2003 onwards, it was not included as a covariate in this analysis but was included in a smaller analysis, of which the results were consistent in direction and magnitude to those in Table S1, suggesting that BMI is not a major explanatory variable.

Another possible proximate explanation for the observed shift in decreased to increased offspring disease and mortality risk is that the mechanisms (and thus subsequent health effects on offspring) underlying early vs. late occurring maternal PIH differ. For example, Yuan et al. [33] suggest that the cascading set of events (i.e. early insufficient shallow cytotrophoblast invasion triggering placental chemical releases that damage maternal endothelium causing increased maternal blood pressure) that ultimately triggers onset of above average maternal blood pressure should not become manifest as clinical symptoms until after trimester 1. This might suggest that the cause of PIH in trimester 1 is not linked to insufficient cytotrophoblast invasion but to a different process that leads, hemodynamically (via blood flow/pressure), to a healthier offspring. PIH in trimester 1 is unlikely to be linked to general blood-pressure problems, as we excluded mothers with any form of pre-existing hypertension. Currently, much remains to be discovered on how the various hypertensive disorders of pregnancy develop and interact throughout pregnancy.

From an evolutionary perspective, the original hypothesis by Haig [12] predicts that POC affects maternal blood pressure throughout pregnancy. Milder forms of PIH would then reflect a balance between fetal factors inducing slightly higher blood pressure (and gaining advantages from these without large maternal costs) and maternal factors decreasing blood pressure. More severe forms of PIH were hypothesized to be due to insufficient blastocyst invasion early in pregnancy (week 20–22, Fig. 1) resulting in conflict over limited resources later in pregnancy, which is more damaging for both mother and offspring, in spite of fetuses also benefitting from increased provisioning [12], [16]. Damaging effects for mother’s own health include increased risks of mortality [34], morbidity [35] and health problems during subsequent pregnancies [36]. Our results suggest that PIH in the first 13 weeks of pregnancy may enhance fetal blastocyst invasion leading to a more accessible maternal blood supply later in pregnancy. The timing of PIH may therefore be linked with Darwinian fitness so that natural selection may be responsible for the maintenance of these disorders in modern humans.

If genetic variation related to POC [12], [20] exists, PIH in trimester 1 might be achieved when imprinted placental genes of both parents are somewhat overexpressed, but without paternal genes imposing challenges that the maternal genes cannot meet so placental growth will be a balanced function of both forces. However, if PIH first occurs later in pregnancy, this may represent resource demands by paternal genes in response to suboptimal placental investment early in pregnancy, which would be consistent with the shift from enhanced to compromised offspring health depending on the timing of PIH. This appears consistent with recent molecular and genomic studies that have discovered or confirmed many genes with parent-of-origin effects on fetal growth [18] and blood pressure [37] and even genes related to invasiveness of the placenta in trimester 1 [38]. Our present results suggest that systematic gene-expression and methylation studies of placentas across a wide range of normal and slightly abnormal pregnancies would be a valuable contribution to understanding the etiology of PIH from a POC perspective, rather than focusing only on mechanistic explanations of the separate syndromes.

In conclusion, our study provides epidemiological evidence of health benefits provided by the milder form of a condition that is generally considered maladaptive, and offers a clearer picture of why hypertensive disorders in pregnancy may continue to persist in modern humans. The results of our study confirm that large public health databases are useful for testing questions inspired by evolutionary theory [39]–[41] and for generating novel questions about molecular developmental pathways and the timing of their expression.

## Materials and Methods

### Study Population

The ongoing Danish National Patient Registry (DNP) has collected and electronically stored all diseases diagnosed within hospitals in the Danish population since 1977. From 1977–1993 all diagnoses were coded by the International Classification of Diseases, eighth revision (ICD-8) and from 1994 onwards by the tenth revision (ICD-10) (http://www.medinfo.dk/sks). Unique personal identification numbers (PNRs also known as anonymized ‘Central Person Register’ or CPR numbers) were used to link the DNP data with a number of other population registers including the Danish Civil Registration System, the Fertility Database and the Cause of Death registry. We obtained approval for our study from the Danish Data Protection Agency (www.datatilsynet.dk), the Danish National Board of Health (www.sst.dk) and Statistics Denmark (www.dst.dk), where the latter was the direct data provider. All data were previously de-identified and hosted on a secure computer by Statistics Denmark.

### The Maternal Sample

We extracted information on all mothers giving birth to singletons from 1 January 1977 to 31 December 2007 (*n* = 1,004,129 mothers, *n* = 1,872,192 births, *n* = 7,488,768 offspring diagnoses recorded since 1977) and excluded twin and higher order births (*n* = 61,180 births). The sample was further reduced to 413,594 after excluding mothers due to: missing values for gestation length in 1977–1978, mismatches between maternal birth-related diagnoses and corresponding births due to short interbirth intervals, birth information erroneously matching with several biological mothers, and having a previous diagnosis of potential impact in PIH or preeclampsia: hypertension, diabetes, hypotension, purpura (purple discolorations under the skin due to inflammation), some circulatory diseases (ischaemic heart diseases; ICD-8/ICD-10 codes 41309, 41009, 41199, 41409/DI20–25) and some diseases of the genitourinary system and liver (insufficient kidney; ICD-8/ICD-10 codes 59309, 59319–59327/DN17, liver functioning; ICD-8/ICD-10 codes 57008–57009/DK72). The final sample (*n* = 413,594 mothers, *n* = 758,524 singleton births, *n* = 3,537,525 offspring diagnoses recorded since 1977, Table 1) included women with a singleton birth who were either diagnosed with pregnancy-induced hypertension (without proteinurea; ICD-8/ICD-10 codes 63700/DO13, DO139; *n* = 9,788 births to 8,607 mothers), or with preeclampsia (ICD-8/ICD-10 codes DO14, 63703/DO140, 63704/DO141, 63709/DO149; *n* = 44,531 births to 38,499 mothers). The remaining cases were normotensive singleton pregnancies (*n* = 704,013 births to 367,709 mothers).

### Women Defined as having Pregnancy-induced Hypertension (Without Proteinuria)

Of the women defined as having pregnancy-induced hypertension (PIH) we included only those with the specific ICD-10 diagnoses DO13 and DO139 (ICD-8 63700, Table S2), but we excluded women from this category if PIH developed into preeclampsia later in pregnancy. PIH can be diagnosed in women who do not have protein in the urine but have a blood pressure in the range of 130–139/85–89 mmHg at any stage during pregnancy.

Denmark has a publicly funded health care system that is freely available to the entire population. Every pregnant Danish woman therefore has approximately three scheduled meetings with her general practitioner (GP) and about six meetings with her midwife. At each meeting her blood pressure is measured and urine samples analysed (with urine dip stick). Since PIH is one of the potential precursors of preeclampsia (but not necessary leading to it), women with PIH are subsequently monitored more frequently by their GP or midwife (except when PIH disappears). This could explain why there is a higher incidence of women with only a few days hypertension and fewer cases with a consistent PIH diagnose. PIH is registered but rarely treated, which suggests that not all women with gestational hypertension are recorded with this diagnosis at the hospital, leading to an unknown amount of under-reporting for PIH. The specificity of the Danish National Patient Registry has been validated as being high in general, but a control study indicated that PIH is indeed underestimated [42]. We note, however, that underreporting will tend to make the outcomes of our analyses conservative as it will make the difference between early PIH and normotensive pregnancies less pronounced.

To assess the risk of PIH on subsequent health of offspring, we created three dummy variables that marked whether PIH occurred in trimester one, two and three (Fig. 2A) and used PNRs to collect information on offspring health (mortality and disease) after birth. While the classification of a pregnancy into a trimester scheme is pragmatic clinical practice, it does not reflect the biological development of a pregnancy very well. For example, during the first few weeks of a pregnancy the fetus invests heavily in initial growth and development of the placenta [12]. During this period, the spiral arteries (fetal blood veins) are forming (Fig. 1), so if strong connections with maternal tissue are made early on (perhaps enhanced via increased blood pressure), this could give the fetus a good start for the rest of pregnancy. In week 8 the fetus weighs around 8 g with the placenta accounting for 85% of this [12]. After week 22 the development of the maternal blood supply to the placenta is completed and at this point in time the fetus starts gaining weight more rapidly and begins to accumulate body fat. In week 38, fetal weight is estimated to be 3,250 grams on average, whereas the placenta now only accounts for just 12% of this (Fig. 1) [12].

### Women Defined as having Preeclampsia

Women were diagnosed with different degrees of preeclampsia (Table S2) that included unspecified preeclampsia (ICD-8/ICD-10 codes 63709/DO149), mild preeclampsia (63703/DO140) or severe preeclampsia (63704/DO141). We grouped these into one preeclampsia category for analysis. To be diagnosed with preeclampsia during pregnancy, women must be measured twice as having a blood pressure ≥140/90 mmHg after 20 weeks of gestation combined with high amounts of protein in the urine (proteinurea) [2]. Severe preeclampsia is defined by a blood pressure ≥160/110 mmHg accompanied by proteinurea, headaches or other cerebral and visual disturbances.

### Disease Classifications Used for Offspring Health

Using the PNR (CPR) numbers from the 758,524 offspring born to 413,594 mothers, we extracted information on offspring mortality (deaths recorded within one year after birth) and diseases these offspring were diagnosed with from birth (years ranged from 1979–2007) until 31 December 2009. Offspring diseases were grouped according to 14 standard disease classifications (Table S1) that the Danish National Board of Health maintains (www.medinfo.dk). These 14 classes capture essentially all diseases that hospitals in Denmark diagnose except for accidents, which we excluded because no relationships with conditions in the womb were to be expected.

### Assessing the Risk of Offspring Mortality and Disease

We used Cox proportional- hazard regression for survival data in R version 2.13.2 to estimate risk of mortality (up to 1 year after birth) and disease (up to 27 years after birth, Table S1) for offspring born from pregnancies that were defined as normotensive, with PIH or preeclampsia (Table S2). Mortality rates beyond year one for offspring born to hypertensive mothers were too low (Fig. 2B) to be able to obtain reliable regression coefficients. We used Kaplan-Meier plots to investigate survival of offspring born to normotensive mothers compared to mothers with PIH in trimester 2 and 3 (Fig. 2C). We excluded offspring who died of accidental causes and diagnoses that did not specifically refer to offspring (obstetric; ICD-8/ICD-10 codes 63000–67809/DO00–99, perinatal; ICD-8/ICD-10 codes 76000–77999/DP00–99, injury and external causes; ICD-8/ICD-10 codes 80000–99999/DS00- DT98). We included a birth cohort variable to account for temporal variation in predictor variables.

### General Procedures for Statistical Analysis

All analyses were adjusted for the following potentially confounding maternal/paternal effects: previous spontaneous or induced abortions, parity (how many live offspring a woman previously gave birth to), average parental age at birth, combined family income, mothers education level. Family income was calculated as the average of general income before taxes (across the years 1979–2007) between the mother and father (also in cases where the parents lived separately). Maternal education was recorded in years, beginning from year 1980. We also included the following covariates to adjust for possible confounding offspring effects: sex of the baby, birth weight and length (adjusted for gestational age), year of birth in five-year cohorts, and Apgar5 score. Apgar5 is a general health evaluation made by the midwife or doctor of the neonate’s skin colour, breathing, reflexes, heartbeat and muscle tone five minutes after birth. Each factor can give two points, so the score is between 0–10, where 10 is given to the most healthy appearing babies. To account for possible parabolic effects, quadratic terms for maternal and paternal age were included, but subsequently removed as these were non-significant in all analyses. Scatterplots of the different variables were used to visualize and exclude any outliers in the dataset. The cut-offs for gestational length were set to a minimum of 140 days (20 weeks) and a maximum of 315 days (45 weeks). The cut-offs for birth weight were set to a minimum of 200 grams and a maximum of 6,500 grams.

Model diagnostic procedures were run for the Cox regressions including checking for violation of the assumption of proportional hazards, for disproportional data, and for nonlinearity in the relationship between the log hazard and the covariates. All are standard tests used to check whether a fitted Cox regression adequately describes the data. Tests for proportional hazards were based on scaled Schoenfeld residuals and indicated no violation of proportional hazards for any of the covariates. Potential outliers were removed after identifying them by comparing dfbeta values to the regression coefficients obtained from the Cox regressions. No covariates displayed significant nonlinearity as confirmed by plotting martingale residuals against covariates from the Cox regression. All significant *P* values were Bonferroni corrected.

## Supporting Information

Table S1 Risk of disease (from birth up to 27 years of age) within the 14 main disease groups depending on whether offspring were born to mothers with PIH (trimester 1, 2 or 3) or preeclampsia (after week 20). Values are Risk Ratios (RR <1 when risk is reduced and RR >1 when risk is increased) from Cox regressions, including their 95% confidence intervals in brackets. *P<0.05, **P<0.01, ***P<0.001. Bolded RR and *P* values indicate significance after Bonferroni correction (*α* = 0.05/14 = 0.0035 ). *^ns^ P* values were also obtained using a resampling procedure in which disease scores were randomly shuffled across individuals in the dataset to obtain a null distribution for each RR where there is no relationship between traits and disease. The *P* values of these analyses refer to the number of times (out of 5000 permutations) in which the RR pseudo-estimate was equal to or less than the originally estimated RR, with *^ns^* indicating significant *P* values obtained from Cox regressions becoming non-significant when estimated from the resampling procedure.
(DOC)

Table S2 List of the maternal pregnancy-related complications (and their ICD codes) used in our study. We grouped the various forms of preeclampsia (# 2–5 below) into one category for results presented in Fig. 3 and Table S1.
(DOCX)

Table S3 Risk of disease (from birth up to 27 years of age) within the 14 main disease groups depending on whether offspring were born to mothers with PIH diagnosed in only a single trimester 1, 2 or 3 (i.e. Fig. 2A, dark grey bars). Values are Risk Ratios (RR <1 when risk is reduced and RR >1 when risk is increased) from Cox regressions, including their 95% confidence intervals in brackets. *P<0.05, **P<0.01, ***P<0.001. Bolded *P* values indicate significance after Bonferroni correction (*α* = 0.05/14 = 0.0035 ).
(DOCX)
