# Supplementary material for: Parent-Offspring Conflict and the Persistence of Pregnancy-Induced Hypertension in Modern Humans
Source: PLoS One. 2013 Feb 25;8(2):e56821. doi: 10.1371/journal.pone.0056821 (PMC3581540; doi:10.1371/journal.pone.0056821)
Supplement: Table S1 — Risk of disease (from birth up to 27 years of age) within the 14 main disease groups depending on whether offspring were born to mothers with PIH (trimester 1, 2 or 3) or preeclampsia (after week 20). Values are Risk Ratios (RR <1 when risk is reduced and RR >1 when risk is increased) from Cox regressions, including their 95% confidence intervals in brackets. *P<0.05, **P<0.01, ***P<0.001. Bolded RR and P values indicate significance after Bonferroni correction (α = 0.05/14 = 0.0035 ). ns P values were also obtained using a resampling procedure in which disease scores were randomly shuffled across individuals in the dataset to obtain a null distribution for each RR where there is no relationship between traits and disease. The P values of these analyses refer to the number of times (out of 5000 permutations) in which the RR pseudo-estimate was equal to or less than the originally estimated RR, with ns indicating significant P values obtained from Cox regressions becoming non-significant when estimated from the resampling procedure. (DOC) [file pone.0056821.s001.doc]

**Table S1**

|  |  |  |  | **pregnancy-induced hypertension (PIH)** | | | **preeclampsia** |
| --- | --- | --- | --- | --- | --- | --- | --- |
| **#** | **ICD-8** | **ICD-10^++^** | **disease description** | **trimester 1** | **trimester 2** | **trimester 3** | **(from week 20)** |
| 1 | 0-13999 | DA00-DB99 | infection including parasites | 1.06 (0.81-1.39) | 1.07 (0.89-1.28) | 1.05 (0.99-1.11) | **1.23 (1.19-1.26)***** |
| 2 | 14000-23999 | DC00-DD48 | neoplasms | 0.58 (0.24-1.39) | 0.97 (0.65-1.43) | 1.12 (1.00-1.25)**^ns^* | **1.11 (1.04-1.18)**** |
| 3 | 28000-28999 | DD50-DD89 | blood and blood-forming organs | 0.51 (0.15-1.76) | 1.03 (0.58-1.82) | 0.97 (0.81-1.17) | **1.24 (1.14-1.35)***** |
| 4 | 24000-27999 | DE00-DE90 | endocrine, nutritional, metabolic | 0.83 (0.53-1.29) | 1.26 (0.97-1.62) | **1.37 (1.26-1.49)***** | **1.57 (1.50-1.64)***** |
| 5 | 29000-31599 | DF00-DF99 | mental and behavioural | 0.82 (0.44-1.52) | 1.17 (0.87-1.58) | 0.99 (0.90-1.09) | **1.27 (1.22-1.33)***** |
| 6 | 32000-35899 | DG00-DG99 | nervous system | 0.61 (0.34-1.09) | 1.45 (1.09-1.91)* | 1.04 (0.94-1.15) | **1.30 (1.24-1.36)***** |
| 7 | 36000-38999 | DH00-DH95 | eye and adnexa | 0.86 (0.65-1.14) | 1.25 (1.06-1.49)* | 1.05 (0.99-1.11) | **1.21 (1.18-1.25)***** |
| 8 | 39000-45899 | DI00-DI99 | circulatory system | 0.60 (0.23-1.57) | 1.18 (0.78-1.79) | **1.28 (1.13-1.45)***** | **1.29 (1.21-1.39)***** |
| 9 | 46000-51999 | DJ00-DJ99 | respiratory system | 0.80 (0.64-0.99)* | 1.08 (0.95-1.24) | 1.04 (1.00-1.09) | **1.25 (1.22-1.28)***** |
| 10 | 52000-57799 | DK00-DK93 | digestive system | 0.88 (0.63-1.22) | 1.06 (0.88-1.27) | 1.01 (0.95-1.07) | **1.23 (1.20-1.27)***** |
| 11 | 68000-70999 | DL00-DL99 | skin and subcutaneous tissue | 0.53 (0.33-0.86)* | 1.17 (0.93-1.47) | 1.10 (1.02-1.19)** | **1.16 (1.12-1.20)***** |
| 12 | 71000-73799 | DM00-DM99 | musculoskeletal, connective tissue | 1.36 (1.00-1.85)* | 0.95 (0.79-1.15) | 1.02 (0.97-1.08) | **1.11 (1.08-1.15)***** |
| 13 | 58000-62999 | DN00-DN99 | genitourinary system | 0.90 (0.61-1.32) | 1.19 (0.97-1.46) | 1.05 (0.98-1.12) | **1.11 (1.08-1.15)***** |
| 14 | 73800-75999 | DQ00-DQ99 | congenital, chromosomal abnormalities | 0.81 (0.60-1.11) | 1.12 (0.93-1.36) | 1.09 (1.02-1.16)* *^ns^* | **1.19 (1.15-1.23)***** |
| *Number of groups with decreased risk (RR < 1)* | | | | *12* | *2* | *2* | *0* |
| *Number of groups with significantly increased risks* | | | | *1* | *2* | *5* | *14* |

**^++^**Classifications of disease groups can be found at www.medinfo.dk/sks, which largely correspond to those found at www.who.int/classifications/icd/en/.
