# Supplementary material for: Parent-Offspring Conflict and the Persistence of Pregnancy-Induced Hypertension in Modern Humans
Source: PLoS One. 2013 Feb 25;8(2):e56821. doi: 10.1371/journal.pone.0056821 (PMC3581540; doi:10.1371/journal.pone.0056821)
Supplement: Table S2 — List of the maternal pregnancy-related complications (and their ICD codes) used in our study. We grouped the various forms of preeclampsia (# 2–5 below) into one category for results presented in Fig. 3 and Table S1. (DOCX) [file pone.0056821.s002.docx]

**Table S2**

| **#** | **ICD-8** | **ICD-10** | **disease description** |
| --- | --- | --- | --- |
| 1 | 63700 | DO13 / DO139 | pregnancy-induced hypertension (PIH) without proteinurea |
| 2 |  | DO14 | preeclampsia |
| 3 | 63703 | DO140 | mild preeclampsia |
| 4 | 63704 | DO141 | severe preeclampsia |
| 5 | 63709 | DO149 | unspecified preeclampsia |
