# Supplementary material for: Parent-Offspring Conflict and the Persistence of Pregnancy-Induced Hypertension in Modern Humans
Source: PLoS One. 2013 Feb 25;8(2):e56821. doi: 10.1371/journal.pone.0056821 (PMC3581540; doi:10.1371/journal.pone.0056821)
Supplement: Table S3 — Risk of disease (from birth up to 27 years of age) within the 14 main disease groups depending on whether offspring were born to mothers with PIH diagnosed in only a single trimester 1, 2 or 3 (i.e. Fig. 2A, dark grey bars). Values are Risk Ratios (RR <1 when risk is reduced and RR >1 when risk is increased) from Cox regressions, including their 95% confidence intervals in brackets. *P<0.05, **P<0.01, ***P<0.001. Bolded P values indicate significance after Bonferroni correction (α = 0.05/14 = 0.0035 ). (DOCX) [file pone.0056821.s003.docx]

**Table S3**

|  |  |  |  | **pregnancy-induced hypertension (PIH)** | | |
| --- | --- | --- | --- | --- | --- | --- |
| **#** | **ICD-8** | **ICD-10^++^** | **disease description** | **trimester 1** | **trimester 2** | **trimester 3** |
| 1 | 0-13999 | DA00-DB99 | infection including parasites | 1.67 (0.70-4.02) | 1.25 (0.88-1.77) | 1.05 (0.99-1.12) |
| 2 | 14000-23999 | DC00-DD48 | neoplasms | 0.00013 (7.81^e-88^-2.29^e+79^) | 1.01 (0.48-2.13) | 1.12 (1.00-1.26)* |
| 3 | 28000-28999 | DD50-DD89 | blood and blood-forming organs | 0.00012 (1.54^e-133^-8.91^e+124^) | 0.96 (0.31-2.96) | 0.97 (0.81-1.16) |
| 4 | 24000-27999 | DE00-DE90 | endocrine, nutritional, metabolic | 1.83 (0.46-7.31) | 1.60 (0.98-2.61) | **1.38 (1.27-1.50)***** |
| 5 | 29000-31599 | DF00-DF99 | mental and behavioural | 0.00012 (9.76^e-69^-1.54^e+60^) | 1.18 (0.70-2.00) | 0.99 (0.90-1.09) |
| 6 | 32000-35899 | DG00-DG99 | nervous system | 0.90 (0.13-6.42) | 0.95 (0.49-1.82) | 1.02 (0.92-1.13) |
| 7 | 36000-38999 | DH00-DH95 | eye and adnexa | 1.76 (0.73-4.22) | 1.32 (0.94-1.87) | 1.05 (0.99-1.11) |
| 8 | 39000-45899 | DI00-DI99 | circulatory system | 0.00013 (1.71^e-105^-9.43^e+96^) | 1.63 (0.82-3.26) | **1.30 (1.14-1.47)***** |
| 9 | 46000-51999 | DJ00-DJ99 | respiratory system | 1.71 (0.89-3.28) | 1.24 (0.96-1.60) | 1.04 (1.00-1.09) |
| 10 | 52000-57799 | DK00-DK93 | digestive system | 0.83 (0.27-2.58) | 1.30 (0.95-1.79) | 1.02 (0.96-1.08) |
| 11 | 68000-70999 | DL00-DL99 | skin and subcutaneous tissue | 0.52 (0.07-3.71) | 1.40 (0.92-2.13) | 1.11 (1.03-1.20)** |
| 12 | 71000-73799 | DM00-DM99 | musculoskeletal, connective tissue | 0.50 (0.13-2.01) | 0.85 (0.59-1.22) | 1.02 (0.96-1.07) |
| 13 | 58000-62999 | DN00-DN99 | genitourinary system | 0.81 (0.20-3.25) | 1.29 (0.89-1.87) | 1.05 (0.98-1.12) |
| 14 | 73800-75999 | DQ00-DQ99 | congenital, chromosomal abnormalities | 0.80 (0.20-3.18) | 0.93 (0.59-1.46) | 1.08 (1.01-1.16)* |
| *Number of groups with decreased risk (RR < 1)* | | | | *10* | *4* | *2* |
| *Number of groups with significantly increased risks* | | | | *0* | *0* | *5* |

**^++^**Classifications of disease groups can be found at www.medinfo.dk/sks, which largely correspond to those found at www.who.int/classifications/icd/en/.
